# GTP binding protein 2 maintains the quiescence, self-renewal, and chemoresistance of mouse colorectal cancer stem cells via promoting Wnt signaling activation

**DOI:** 10.1016/j.heliyon.2024.e27159

**Published:** 2024-03-01

**Authors:** Chao Ke, Hongjian Zhou, Tian Xia, Xingwang Xie, Bin Jiang

**Affiliations:** The Department of Gastrointestinal, Hernia and Abdominal Wall Surgery, Wuhan Third Hospital (Tongren Hospital of Wuhan University), 241 Pengliuyang Road, Wuchang District, Wuhan, Hubei Province, 430060, China

**Keywords:** Colorectal cancer stem cells, GTP binding protein 2, Proliferation, Self-renewal, Chemoresistance, Stemness

## Abstract

Colorectal cancer (CRC) is one of the most common cancers and the second most deadly cancer across the globe. Colorectal cancer stem cells (CCSCs) fuel CRC growth, metastasis, relapse, and chemoresistance. A complete understanding of the modulatory mechanisms of CCSC biology is essential for developing efficacious CRC treatment. In the current study, we characterized the expression and function of GTP binding protein 2 (GTPBP2) in a chemical-induced mouse CRC model. We found that GTPBP2 was expressed at a higher level in CD133^+^CD44^+^ CCSCs compared with other CRC cells. Using a lentivirus-based Cas9/sgRNA system, GTPBP2 expression was ablated in CRC cells *in vitro*. GTPBP2 deficiency caused the following effects on CCSCs: 1) Significantly accelerating proliferation and increasing the proportions of cells at G1, S, and G2/M phase; 2) Impairing resistance to 5-Fluorouracil; 3) Weakening self-renewal but not impacting cell migration. In addition, GTPBP2 deficiency remarkably decreased β-catenin expression while increasing β-catenin phosphorylation in CCSCs. These effects of GTPBP2 were present in CCSCs but not in other CRC cell populations. The Wnt agonist SKL2001 completely abolished these changes in GTPBP2-deficient CCSCs. When GTPBP2-deficient CCSCs were implanted in nude mice, they exhibited consistent changes compared with GTPBP2-expressing CCSCs. Collectively, this study indicates that GTPBP2 positively modulates Wnt signaling to reinforce the quiescence, self-renewal, and chemoresistance of mouse CCSCs. Therefore, we disclose a novel mechanism underlying CCSC biology and GTPBP2 could be a therapeutic target in future CRC treatment.

## Abbreviations

CRC: Colorectal cancer
CCSCs: Colorectal cancer stem cells
GTPBP2: GTP binding protein 2
EpCAM: Epithelial cell adhesion molecule
CK1: Casein kinase 1
GSK-3β: Glycogen synthase 3β
APC: Adenomatous polyposis coli
OCT4: Octamer-binding transcription factor 4
SOX2: SRY-box 2
NANOG: Nanog Homeobox.

## 1 Introduction

Colorectal cancer stem cells (CCSC) are crucial for the initiation, progression, recurrence, and metastasis of colorectal cancer (CRC). Their self-renewal maintains the CCSC pool and they can generate all relevant cancer cells with various differentiation statuses [1]. CCSCs are likely in a relatively quiescent state, making them resistant to radiochemotherapy [1]. Until recently, several cell surface proteins, such as CD133, CD44, epithelial cell adhesion molecule (EpCAM), and CD24, have been identified as CCSC-related markers [2]. Particularly, CD133^+^CD44^+^ CRC cells were considered as CCSCs owing to their cancer-initiating ability [[3], [4], [5]]. However, the distribution of CCSC markers differs between patients and cell lines. Some studies argue that CD44^+^CD133^−^ cells have CCSC features while CD133^high^CD24^low^ tumors correlate with the worst prognosis in patients [[6], [7], [8]]. Therefore, the genuine CCSC markers remain elusive.

The canonical Wnt signaling is important for CRC development. Without Wnt ligands, intracellular β-catenin is phosphorylated by a destruction complex consisting of Casein kinase 1 (CK1), glycogen synthase 3β (GSK-3β), Axin, and adenomatous polyposis coli (APC). Phosphorylated β-catenin is easily degraded and thus kept at a low level. When Wnt ligands bind to the Frizzled receptor, β-catenin is released from the destruction complex to avoid degradation and subsequently enters the nucleus to trigger the transcription of target genes such as c-myc, c-jun, survivin, and OCT4, etc [9]. Cumulative evidence has demonstrated aberrant activation of Wnt signaling as an indispensable factor in CRC carcinogenesis [10]. Particularly, Wnt signaling is critical for maintaining stemness, chemoresistance, and metastasis of CCSCs [[11], [12], [13], [14]]. Therefore, understanding the regulatory mechanisms of Wnt signaling is critical for studying CCSC biology.

GTP-binding proteins are proteins with GTP hydrolase activity and participate in cell signal transduction, cytoskeletal rearrangement, gene expression, and other biochemical processes. GTP binding protein 2 (GTPBP2) is a member of the G protein superfamily and is expressed in most organs and tissues, especially in the cerebral cortex, cerebellum, thymus, smooth muscle, liver, pancreas, and digestive tract [15,16]. Recent research reports that GTPBP2 is a positive regulator of the invasion, migration, and proliferation of non-small cell lung cancer [17]. Notably, GTPBP2 is necessary for canonical Wnt signaling in *Xenopus* embryos, via suppressing the accumulation of Axin, which is a component of the β-catenin destruction complex [18]. Since Wnt signaling is crucial for CCSC development, GTPBP2 likely regulates CCSC biology by modulating Wnt signaling. However, the significance of GTPBP2 to the development of CCSCs has not been disclosed. In this research, we characterized the role of GTPBP2 in regulating CCSC proliferation, self-renewal, and chemoresistance in a chemical-induced primary CRC model.

## 2 Materials and methods

### 2.1 Primary CRC model

This animal research was approved by the Wuhan Third Hospital Animal Care and Use Committee (Approval ID: WTH20210146) and conducted under the Animal Research: Reporting of In Vivo Experiments (ARRIVE) guidelines. Eight-week-old male C57BL/6J mice were purchased from Wanqian Animal Technology Inc. Azoxymethane (AOM, Cat# A5486) and dextran sulfate sodium (DSS, Cat# D8906-50G, molecular weight = 36–50 kDa) were purchased from Sigma-Aldrich. AOM was diluted in sterile saline at a concentration of 2.5 mg/ml. The model was established according to previous reports with minor modifications [19,20]. Briefly, mice were injected intraperitoneally with 10 mg AOM per kg mouse weight. After 6 days, mice were treated with three cycles of 2% DSS for 7 days in drinking water, followed by 2 weeks of regular drinking water (Supplementary Fig. 1). Three months after the AOM injection, mice were euthanized with CO_2_ and tumor formation was assessed by gross examination. Mice with visible tumor(s) in the colon and rectum were used in the study (Supplementary Fig. 1). The control mice did not receive AOM injection and were fed with regular drinking water.

### 2.2 Isolation of tumor cells from primary CRC tissues

According to previous reports with modifications [21,22], the tumors were taken from mouse colons and rectums, minced into 1-mm^3^ pieces and incubated in EDTA buffer (2 mM EDTA, 43.4 mM sucrose, 54.9 mM D-sorbitol, 0.5 mM DL-dithiothreitol in PBS) for 60 min on ice. After two washes with PBS, the tissues were dissociated in digestion buffer (DMEM supplemented with 5% fetal calf serum, 2.5 ng/ml of amphotericin B, 200 U/ml collagenase IV, and 0.1 mg/ml type II dispase) for 45 min at 37 °C with agitation every 15 min. The supernatants and tissues were filtered through a 70-μm cell strainer to prepare single cells, followed by centrifugation at 200*g* for 3 min and washing once with 5 ml of PBS before further experiments. The reagents were purchased from Sigma-Aldrich.

### 2.3 Flow cytometry

PE/Cy7 anti-CD133 (315-2C11), APC-Cy7 anti-CD44 (IM7), FITC anti-CD45 (QA17A26), FITC anti-CD31 (390), and Pacific Blue anti-Ki67 (11F6) were purchased from BioLegend. The polyclonal anti-MCT4 antibody (22787-1-AP), FITC goat anti-rabbit IgG (F-2765), PE goat anti-rabbit IgG (A10542), anti-phospho-β-catenin (Ser33) polyclonal antibody (BS-12583R), PE anti-β-catenin antibody (15B8), eFluor™ 660 anti-SOX2 antibody (Btjce), eFluor™ 660 anti-OCT4 antibody (EM92), and eFluor™ 660 anti-NANOG antibody (eBioMLC-51) were obtained from ThermoFisher. For surface marker detection, 1 × 10^6^/ml cells were incubated with 5 μg/ml of each antibody for 30 min on ice. For intracellular protein detection, cells were fixed in 200 μl of Cytofix/Cytoperm solution (BD Biosciences) for 20 min on ice. Cells were then permeabilized in 200 μl of 90% methanol-PBS for 30 min at 4 °C, followed by staining with 2 μg/ml antibodies for 1 h at room temperature. When a primary antibody was unconjugated, the cells were washed with PBS once and incubated with 5 μg/ml fluorophore-conjugated antibody for 30 min on ice. For apoptosis analysis, cells were incubated with an APC Annexin V Apoptosis Detection Kit with propidium iodide (640932, Biolegend) according to the manufacturer's instructions. Cells were analyzed on a BD FACSCalibur™ cytometer (BD Biosciences). Cell sorting was performed on a BD Influx cell sorter (BD Biosciences).

### 2.4 Lentivirus preparation and transduction

The Lenti-Cas9-gRNA-GFP vector (Cat# 124770) encoding Cas9, sgRNA, and green fluorescence protein (GFP) was purchased from Addgene. The *Gtpbp2* sgRNA (5′-ATTATGACAGTGACGTGCCC-3′) and scrambled sgRNA (5′-GCACTACCAGAGCTAACTCA-3′) were designed by Integrated DNA Technologies. Predicted off-target genes of the *Gtpbp2* sgRNA are *Thrb*, *Cndp1*, *Plaa,* and *Fer*. However, we did not observe alterations of mRNAs of these genes in our pilot experiments (Data not shown), suggesting that the *Gtpbp2* gene is the only target. Vector cloning and lentivirus packaging/titration were achieved by AtaGenix Biotech Inc. To infect primary CRC cells, CRC cells were suspended in DMEM supplemented with 10% fetal calf serum (FCS). Cells were seeded in 6-well culture plates at a density of 5 × 10^4^ cells/well. After that, polybrene (Maokang Biotech) was added at a final concentration of 6 μg/ml. Lentiviral particles were added at a multiplicity of transduction (MOI) of 50, followed by centrifugation at 700*g* for 1 h at 32 °C and then incubated overnight. The supernatants were replaced with fresh 10% FCS-DMEM to incubate cells for 2 days. The infection efficiency was determined by assessing the frequency of GFP^+^ cells (i.e. successfully infected cells). GFP^+^ cells were sorted by flow cytometry, followed by detecting their phenotype and function.

To determine the effect of Wnt signaling, sorted GFP^+^ CCSCs were cultured at a density of 5 × 10^5^/ml in DMEM supplemented with 10% FCS. SKL2001 (also known as Wnt agonist II, Cat# 681667, Sigma-Aldrich) was added into the culture at a final concentration of 20 μM to incubate cells for 24 h. Detection of phosphorylated β-catenin, total β-catenin, and Ki67 was then performed by flow cytometry.

To overexpress GTPBP2, GTPBP2 lentiviral vector (22819065) and the control pLenti-GIII-CMV vector (LV587) were purchased from Applied Biological Materials. Lentivirus preparation and infection procedures were the same as above. Two days after infection, the cells were treated with 2 μg/ml puromycin (Sigma-Aldrich) for 24 h to select infected cells. Viable cells were subjected to further analysis.

### 2.5 Cell cycle

Cells were fixed in ice-cold 70% ethanol for 2.5 h, followed by incubation in Hank's balanced salt solution (HBSS) containing 2 mg Hoechst 33342 (94403, Sigma-Aldrich) and 4 mg Pyronin Y (P9172, Sigma-Aldrich) for 20 min. Afterward, cells were washed with PBS once and the intensities of Hoechst 33342 and Pyronin Y were analyzed by flow cytometry.

Sphere formation.

CRC cells were suspended at a density of 2.5 × 10^3^/ml in DMEM/F12 supplemented with 1 × N2 supplement, 1 × B27 supplement, 1 mM N-acetylcysteine, 10 ng/ml EGF, 20 ng/ml basic FGF, and 1 mg/ml heparin (All from Stem Cell Technologies). 1 ml of cell suspension was placed in each well of a 6-well ultra-low attachment culture plate (Corning) and cultured for 7 days to form spheres (Passage 1). The spheres were digested with 0.5% Trypsin-EDTA (Sigma-Aldrich) for 5 min at 37 °C, followed by vigorous pipetting to prepare single cells. The single cells were pelleted by centrifugation and cultured in supplemented DMEM/F12 in the same manner as above to form passage-2 spheres. The above procedures were repeated 7 days later to generate passage-3 spheres. To count the spheres, the culture was centrifuged at 150 *g* for 5 min, followed by aspirating the supernatant and gently re-suspending the sphere pellet in 500 μl of DMEM/F12. After that, 100 μl of the sphere suspension was added into a well (marked with four quadrants on its underside) of a 96-well plate. The spheres (>50 μm in diameter) in each quadrant were counted and summed on a Zeiss AXIO Imager.M1 phase contrast motorized microscope (Zeiss). To determine the effect of Wnt signaling, 20 μM SKL2001 was added at the start of passage-2 sphere culture and was present for 7 days.

### 2.6 Cell migration

5 × 10^4^ CRC cells were suspended in serum-free DMEM and seeded on the upper compartment of Matrigel-coated invasion chambers (Corning) with an 8-μm pore polycarbonate membrane. The lower compartment was filled with 10% FCS-DMEM. After 24-h incubation, cells on the upper side of the membrane were removed using a cell scraper. Migrated cells on the lower side of the membrane were fixed with 95% ethanol, stained with 0.1% crystal violet solution, and counted with a light microscope.

### 2.7 Cell growth and chemoresistance

To evaluate cell growth, 1.25 × 10^3^ CRC cells (in 200 μl RPMI 1640) were cultured in each well of 96-well plates for indicated periods. Cells were rinsed with 10 mM PBS and incubated in 20 μl 3-[4,5-dimethylthiazole-2-yl]-2,5-diphenyltetrazolium bromide (MTT, 5 μg/μl) for 4 h. After twice washes with 10 mM PBS, cells were incubated with 150 μl dimethyl sulfoxide for 10 min. Absorbance at 490 nm was measured on a SpectraMax® ABS Plus microplate reader (Molecular Devices). In chemoresistance analysis, CRC cells were seeded at a density of 5 × 10^3^ cells per well and treated with 5-Fluorouracil (5-FU) at the indicated concentrations for 48 h, followed by the MTT assay as described above. The percentage of cell viability was computed using the equation: % Viability = Mean OD_sample_/Mean OD_blank_ × 100.

Sorted GFP^+^ CRC cells were suspended in 10% FCS-DMEM and seeded in triplicate in 96-well plates at 5 × 10^3^ cells/well, flowed by exposure to 10 μM 5-FU for 48 h. Apoptosis and necrosis were analyzed by flow cytometry. To evaluate the relationship between Wnt signaling and GTPBP2, SKL2001 was added into the culture at 20 μM.

### 2.8 Immunoblotting

Proteins were extracted by incubating cells in the RIPA lysis buffer (Beyotime Inc) containing protease inhibitors (Beyotime Inc) for 30 min on ice. A total of 10 μg whole-cell protein was loaded per lane. The anti-GTPBP2 polyclonal antibody (Cat# PA5-75936, 1:1000) and anti-GAPDH monoclonal antibody (Cat# MA1-16757, 1:2000) were purchased from ThermoFisher. The signals were detected and recorded by a Biospetrum 300 system (UVP Ltd).

### 2.9 Tumor implantation

Eight-to-ten-week-old nude mice were purchased from Wanqian Animal Technology Inc. 1 × 10^6^ CSCs were mixed with 50 μl of Matrigel matrix (Corning) and injected into the right flank. Six weeks later, the mice were euthanized by CO_2_ and the volumes of subcutaneous tumors were measured as (length × width^2^) × 0.5. Following that, tumor cells were isolated as previously described.

### 2.10 Hematoxylin and eosin staining

Normal mouse colon, CRC tumors, and tumor implants were fixed in formalin, embedded in paraffin, and cut into 10-μmm sections following the routine procedures. Hematoxylin and eosin staining was carried out following the standard protocol.

### 2.11 Immunohistochemistry

Tissue sections were prepared as described above. The sections were blocked with 10% normal goat serum (ThermoFisher), followed by incubation with the anti-GTPBP2 polyclonal antibody (Cat# PA5-75936, 1:100) overnight at 4 °C before incubation with the horseradish peroxidase-labeled goat anti-rabbit IgG (31460, Invitrogen) for 1 h at room temperature. After PBS washes, the DAB substrate kit (ab64238, Abcam) was used for detection. The samples were observed on an AXIO Imager Microscope (Zeiss).

### 2.12 RNA purification and real-time PCR

Cellular RNAs were purified using the PureLink RNA Mini Kit (Invitrogen). Tissue RNAs were extracted using TRIzol (ThermoFisher) following the manufacturer's instructions. cDNAs were synthesized using the High-Capacity cDNA Reverse Transcription Kit (Applied Biosystems). The PowerUp™ SYBR™ Green Master Mix (Applied Biosystems) was used to quantify gene expression on a 7300 real-time PCR platform (Applied Biosystem). The expression of target genes was normalized to β-actin and calibrated using the 2^−ΔΔCt^ method. Primers are shown in Supplementary Table 1.

### 2.13 Statistics

All data are presented as mean ± SD. Student's *t*-test or One-way ANOVA was applied to compare mean values among different groups. The GraphPad Prism 9 was used for statistical analysis. All experiments were repeated two or three times. A *P* value < 0.05 is considered statistically significant.

## 3 Results

### 3.1 GTPBP2 is highly expressed in CSCs of primary CRC

**Fig. 1:**
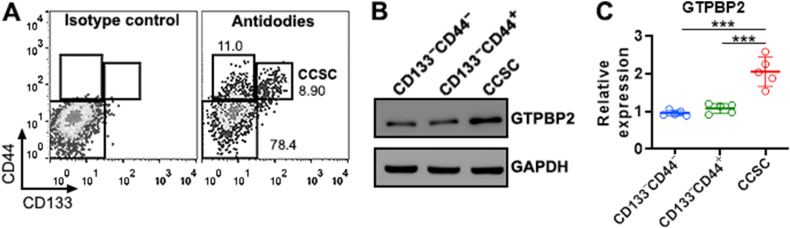
GTPBP2 expression in primary CRC cells. (A) Representative dot plots showing CRC cell subsets. CCSC: colorectal cancer stem cells. **(B and C)** GTPBP2 expression in sorted CRC cell subsets. Representative Immunoblotting images are shown in (B) and statistics are shown in (C). N = 5 independent samples per group. Each sample contains cells pooled from five mice. ***: *P* < 0.001. One-way ANOVA.

To determine GTPBP2 expression in CRC, we established a chemical-induced CRC model. The colon tissue in the AOM/DSS group exhibited loss of goblet cells and epithelial cell destruction, indicating CRC formation (Supplementary Fig. 1). GTPBP2 was moderately expressed in granular cells of normal colons but strongly expressed in CRC cells after AOM/DSS treatment (Supplementary Fig. 2A). The relative mRNA level of GTPBP2 in the CRC tissue negatively correlated with mouse body weight and positively correlated with mean tumor volume but did not correlate with either tumor number or colon length (Supplementary Figs. 2B–2E), suggesting that GTPBP2 might enhance CRC growth. CRC tumors were harvested from the colon and rectum of each mouse, followed by enzyme digestion and mechanical dissociation. The resultant single cells were subjected to flow cytometry analysis. CD45^+^ leukocytes, CD31^+^ vascular endothelial cells, MCT4^+^ stromal cells, and propidium iodide-positive dead cells were excluded (Supplementary Fig. 3). The rest cells, i.e. CRC cells, were divided into subsets based on the expression of CD133 and CD44: CD133^−^CD44^−^, CD133^−^CD44^+^, CD133^+^CD44^+^, and minor CD133^+^CD44^−^ cells (Fig. 1A). CD133^−^CD44^−^, CD133^−^CD44^+^, and CD133^+^CD44^+^ cells were sorted by flow cytometry (Supplementary Fig. 4). Only CD133^+^CD44^+^ cells produced abundant tumor spheres *in vitro*, suggesting that they were CCSCs (Supplementary Fig. 5A). Next, GTPBP2 expression was determined in each subset. CCSCs expressed significantly higher GTPBP2 than CD133^−^CD44^−^ and CD133^−^CD44^+^ cells (Fig. 1B and C and Supplementary Fig. 5B).

### 3.2 *Gtpbp2* knockout down-regulates stemness markers in CCSCs

**Fig. 2:**
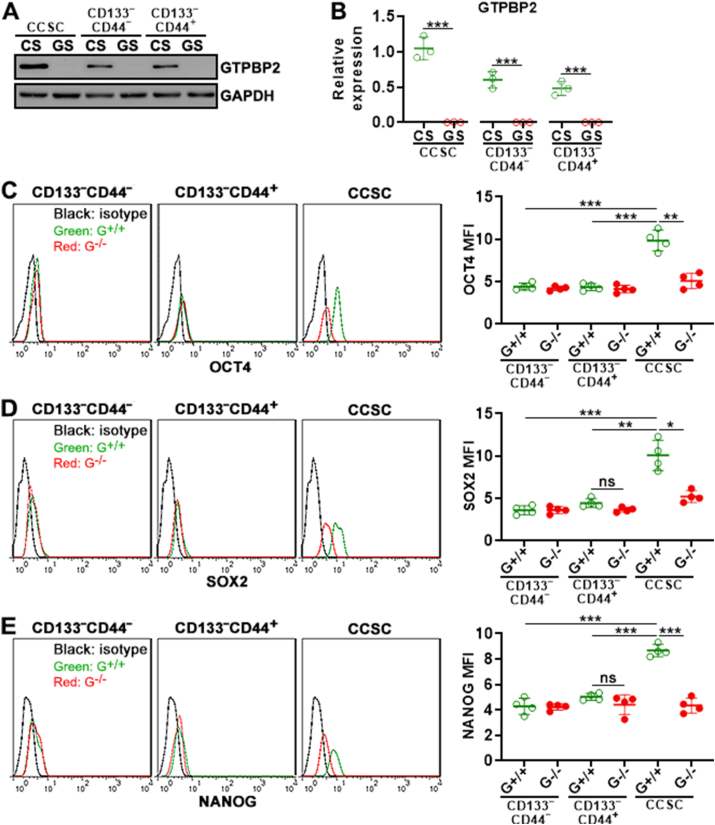
Lentivirus-mediated *Gtpbp2* knockout in primary CRC cells. (A) GTPBP2 protein in GFP^+^ cells of CRC cell subsets on post-infection day 2. CS: control lentivirus encoding a scrambled sgRNA. GS: lentivirus encoding the *Gtpbp2* sgRNA. CCSC: colorectal cancer stem cells. **(B)** Statistics of GTPBP2 protein in GFP^+^ cells. **(C to E)** OCT4 (C), SOX2 (D), and NANOG (E) expression in GFP^+^ cells of CRC cell subsets on post-infection day 2. Left panels: representative histograms. Right panels: Statistics of the mean fluorescence of each marker. Isotype: isotype control. G^+/+^: *Gtpbp2*^+/+^ cells. G^−/−^: *Gtpbp2*^−/−^ cells. N = 3 or 4 independent samples (**no replicate**s) per group. Student's t-test for (B) and One-way ANOVA for (C to E). ns: not significant.

To explore the function of GTPBP2, the *Gtpbp2* gene was ablated in sorted CD133^−^CD44^−^ cells, CD133^−^CD44^+^ cells, and CCSCs using an all-in-one Cas9/sgRNA lentiviral system (Supplementary Fig. 6A). After lentivirus infection, GFP^−^ cells were discarded and GFP^+^ cells (i.e. infected cells) were sorted for analysis (Supplementary Figs. 6B and 6C). No significant differences in apoptosis and necrosis were seen between *Gtpbp2*^−/−^ cells and *Gtpbp2*^+/+^ cells (Supplementary Fig. 6D), suggesting that *Gtpbp2* knockout did not trigger cell death. Immunoblotting demonstrated the absence of GTPBP2 protein in GFP ^+^ cells infected with the *Gtpbp2* sgRNA-encoding lentivirus (Fig. 2A and B and Supplementary Fig. 6E). Therefore, these cells were termed *Gtpbp2*^−/−^ cells, whereas GFP^+^ cells infected with the scramble sgRNA-encoding lentivirus were termed *Gtpbp2*^+/+^ cells.

Next, we measured the impact of *Gtpbp2* knockout on the expression of OCT4, SOX2, and NANOG, all of which are well-known stemness markers [23]. As indicated in Fig. 2C–E, the expression of OCT4, SOX2, and NANOG was moderate in CD133^−^CD44^−^ cells and CD133^−^CD44^+^ cells. Notably, compared with *Gtpbp2*^+/+^ CD133^−^CD44^−^ cells and *Gtpbp2*^+/+^ CD133^−^CD44^+^ cells, *Gtpbp2*^−/−^ CD133^−^CD44^−^ cells and *Gtpbp2*^−/−^ CD133^−^CD44^+^ cells expressed equivalent OCT4, SOX2, and NANOG, respectively. Therefore, GTPBP2 might not modulate the expression of these markers in either CD133^−^CD44^−^ cells or CD133^−^CD44^+^ cells. In contrast, OCT4, SOX2, and NANOG were highly expressed in CCSCs. Importantly, *Gtpbp2*^−/−^ CCSCs expressed remarkably lower OCT4, SOX2, and NANOG than *Gtpbp2*^+/+^ CCSCs, implying that GTPBP2 up-regulated stemness markers in CCSCs.

### 3.3 *Gtpbp2* knockout accelerates CCSC proliferation *in vitro*

**Fig. 3:**
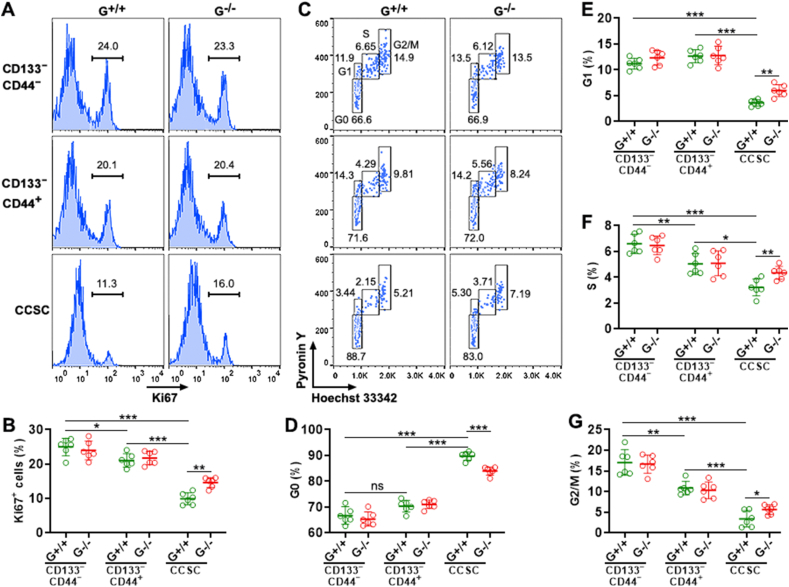
The effect of *Gtpbp2* knockout on CRC cell proliferation. (A and B) Ki67 expression in *Gtpbp2*^+/+^ and *Gtpbp2*^−/−^ CRC cell subpopulations. Representative histograms are shown in (A). Statistics of the frequencies of Ki67^+^ cells are shown in (B). I: subset I. II: subset II. CCSC: colorectal cancer stem cells. G^+/+^: *Gtpbp2*^+/+^ cells. G^−/−^: *Gtpbp2*^−/−^ cells. **(C to G)** Hoechst 33342 and Pyronin Y staining showing the cell cycle status. Representative dot plots are shown in (C). Statistics of the frequencies of cells at each cell cycle phase are shown in (D to G). N = 6 independent samples per group. *: *P* < 0.05. **: *P* < 0.01. ***: *P* < 0.001. One-way ANOVA.

To determine the role of GTPBP2 in regulating CRC cell proliferation, we checked Ki67 expression in CRC cells. 25% of *Gtpbp2*^+/+^ CD133^−^CD44^−^ cells, 20% of *Gtpbp2*^+/+^ CD133^−^CD44^+^ cells, and 11% of *Gtpbp2*^+/+^ CCSCs expressed Ki67, suggesting that CCSCs were relatively quiescent compared to the other two subsets (Fig. 3A and B). *Gtpbp2*^+/+^ CD133^−^CD44^−^ cells and *Gtpbp2*^−/−^ CD133^−^CD44^−^ cells expressed comparable Ki67, suggesting that *Gtpbp2* knockout did not alter the growth of CD133^−^CD44^−^ cells. This was also the case for CD133^−^CD44^+^ cells. In contrast, *Gtpbp2*^−/−^ CCSCs expressed higher Ki67 (15%) than *Gtpbp2*^+/+^ CCSCs (Fig. 3A and B). Therefore, *Gtpbp2* knockout accelerated CCSC expansion. MTT-based growth analysis also indicated that *Gtpbp2*^−/−^ CCSCs grew faster than *Gtpbp2*^+/+^ CCSCs, whereas *Gtpbp2* knockout did not alter the growth of CD133^−^CD44^−^ cells and CD133^−^CD44^+^ cells (Supplementary Fig. 7).

Furthermore, Hoechst 33342 and Pyronin Y were used to evaluate the cell cycle status of CRC cells. As demonstrated in Fig. 3C–G, nearly 70% of *Gtpbp2*^+/+^ CD133^−^CD44^−^ cells and *Gtpbp2*^+/+^ CD133^−^CD44^+^ cells were at G0 phase, whereas 90% of *Gtpbp2*^+/+^ CCSCs were at G0 phase. 11%∼12% of *Gtpbp2*^+/+^ CD133^−^CD44^−^ cells and *Gtpbp2*^+/+^ CD133^−^CD44^+^ cells were at G1 phase, whereas less than 5% of *Gtpbp2*^+/+^ CCSCs were at G1 phase. 7% of *Gtpbp2*^+/+^ CD133^−^CD44^−^ cells and 5% of CD133^−^CD44^+^ cells were at S phase, whereas 3% of *Gtpbp2*^+/+^ CCSCs were at S phase. 16% of *Gtpbp2*^+/+^ CD133^−^CD44^−^ cells and 10% of CD133^−^CD44^+^ cells were at G2/M phase, whereas only 5% of *Gtpbp2*^+/+^ CCSCs were at G2/M phase. Therefore, CCSCs proliferated slower than the other two subsets. *Gtpbp2* knockout did not profoundly change the cell cycle status of CD133^−^CD44^−^ cells and CD133^−^CD44^+^ cells. However, compared with *Gtpbp2*^+/+^ CCSCs, *Gtpbp2*^−/−^ CCSCs contained fewer G0-phase cells and more G1-phase, S-phase, and G2/M-phase cells (Fig. 3C–G), suggesting that GTPBP2 decelerated CCSC expansion. To confirm these results, we assessed the mRNA levels of Cyclins that drive cell cycle progression [24]. As shown in Supplementary Fig. 8, *Gtpbp2*^+/+^ CD133^−^CD44^−^ cells and *Gtpbp2*^+/+^ CD133^−^CD44^+^ cells expressed equivalent Cyclin D1. However, *Gtpbp2*^+/+^ CD133^−^CD44^−^ cells expressed higher Cyclin E1, Cyclin A1, and Cyclin B1 than *Gtpbp2*^+/+^ CD133^−^CD44^+^ cells. Notably, CCSCs expressed the lowest Cyclin D1, Cyclin E1, Cyclin A1, and Cyclin B1 among all three subsets. *Gtpbp2*^−/−^ CD133^−^CD44^−^ cells and *Gtpbp2*^−/−^ CD133^−^CD44^+^ cells expressed equivalent Cyclin D1, Cyclin E1, Cyclin A1, and Cyclin B1 compared to their *Gtpbp2*^+/+^ counterparts, suggesting that GTPBP2 was not essential for cell cycle progression in CD133^−^CD44^−^ cells and CD133^−^CD44^+^ cells. However, *Gtpbp2*^−/−^ CCSCs expressed higher Cyclins than *Gtpbp2*^+/+^ CCSCs, suggesting that GTPBP2 suppressed CCSC proliferation.

### 3.4 *Gtpbp2* knockout impairs CCSC chemoresistance and self-renewal

**Fig. 4:**
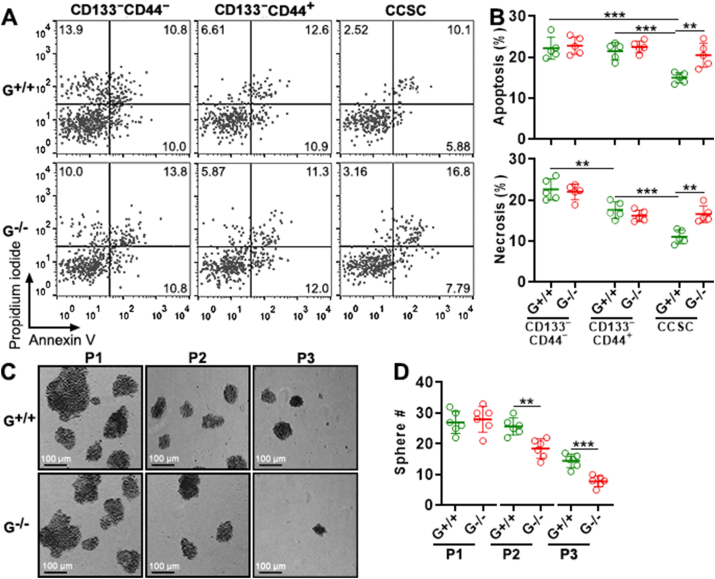
The effect of *Gtpbp2* knockout on chemoresistance, migration, and self-renewal of CRC cells. (A and B) Apoptosis and necrosis of *Gtpbp2*^+/+^ and *Gtpbp2*^−/−^ CRC cell subsets after 48-h 5-FU treatment. Representative dot plots are in (A) and statistics are in (B). G^+/+^: *Gtpbp2*^+/+^ cells. G^−/−^: *Gtpbp2*^−/−^ cells. **(C and D)** Sphere formation by *Gtpbp2*^+/+^ and *Gtpbp2*^−/−^ CCSCs. Representative tumor sphere images are in (C). Statistics of sphere numbers are in (D). P1: passage 1. P2: passage 2. P3: passage 3. Original magnification: 50 × . N = 5 independent samples per group. **: *P* < 0.01. ***: *P* < 0.001. One-way ANOVA.

*Gtpbp2*^−/−^ cells and *Gtpbp2*^+/+^ cells were treated with 5-FU for 48 h to assess their chemosensitivity. 5-FU decreased the viability of *Gtpbp2*^+/+^ CD133^−^CD44^−^ cells (IC50 = 11.73 μM), *Gtpbp2*^+/+^ CD133^−^CD44^+^ cells (IC50 = 14.08 μM), and *Gtpbp2*^+/+^ CCSCs (IC50 = 18.62 μM) in a dose-dependent manner (Supplementary Fig. 9). For CD133^−^CD44^−^ cells and CD133^−^CD44^+^ cells, the *Gtpbp2*^+/+^ group and the *Gtpbp2*^−/−^ group exhibited comparable viability, suggesting that GTPBP2 was not crucial for their chemosensitivity*.* However, *Gtpbp2*^−/−^ CCSCs showed lower viability (IC50 = 14.47 μM) than *Gtpbp2*^+/+^ CCSCs after 10–50 μM 5-FU treatment (Supplementary Fig. 9). Accordingly, GTPBP2 maintained CCSC chemoresistance. We also checked cell apoptosis and necrosis after treatment with 10 μM 5-FU. Compared with *Gtpbp2*^+/+^ CD133^−^CD44^−^ cells and *Gtpbp2*^+/+^ CD133^−^CD44^+^ cells, fewer apoptotic and necrotic cells were observed in *Gtpbp2*^+/+^ CCSCs (Fig. 4A to B), suggesting that CCSCs were more chemoresistant. *Gtpbp2* knockout did not remarkably affect the frequencies of apoptotic and necrotic cells in either CD133^−^CD44^−^ cells or CD133^−^CD44^+^ cells. In contrast, *Gtpbp2*^−/−^ CCSCs had more apoptotic and necrotic cells than *Gtpbp2*^+/+^ CCSCs (Fig. 4A to B). Therefore, GTPBP2 boosted CCSC chemoresistance.

The Transwell assay showed no significant difference in migrating cell number between *Gtpbp2*^−/−^ cells and *Gtpbp2*^+/+^ cells (Supplementary Fig. 10). To appraise the role of GTPBP2 in CCSC self-renewal, the serial sphere formation assay was conducted. At passage 1, *Gtpbp2*^+/+^ CCSCs and *Gtpbp2*^−/−^ CCSCs formed equal numbers of spheres. However, at passage 2 and 3, *Gtpbp2*^−/−^ CCSCs generated fewer spheres than *Gtpbp2*^+/+^ CCSCs (Fig. 4C and D), suggesting GTPBP2 maintained CCSC self-renewal.

### 3.5 *Gtpbp2* overexpression fails to induce the dedifferentiation of CD133^−^CD44^−^ and CD133^−^CD44^+^ CRC cells

To test whether *Gtpbp2* overexpression induces CRC cells to dedifferentiate into CCSCs, we infected CD133^−^CD44^−^ and CD133^−^CD44^+^ cells with a GTPBP2-encoding lentivirus. After puromycin selection, Immunoblotting confirmed *Gtpbp2* overexpression in infected cells (Supplementary Fig. 11A). Surprisingly, *Gtpbp2* overexpression did not up-regulate OCT4, SOX2, and NANOG in infected cells (Supplementary Fig. 11B). Besides, *Gtpbp2* overexpression did not affect Ki67 expression (Supplementary Fig. 11C) or the viability of these cells after 5-FU treatment (Supplementary Fig. 11D). Therefore, GTPBP2 seemed irrelevant to stemness of CD133^−^CD44^−^ and CD133^−^CD44^+^ cells.

### 3.6 GTPBP2 is dispensable for the function of the minor CD133^+^CD44^−^ CRC subset

CD133^+^CD44^−^ CRC cells accounted for only 2% of total CRC cells (Fig. 1A). A previous study states that CD133^+^CD44^−^ cells are putative CCSCs [8]. Accordingly, we checked the features of CD133^+^CD44^−^ cells. The mRNA levels of GTPBP2, OCT4, SOX2, and NANOG in CD133^+^CD44^−^ cells were equivalent to those in CD133^−^CD44^−^ and CD133^−^CD44^+^ cells (Supplementary Figs. 12A–12D). Furthermore, CD133^+^CD44^−^ cells could not generate spheres *in vitro* (Supplementary Fig. 12E), suggesting that they were not CCSCs. After infection with the Cas9/sgRNA lentivirus, *Gtpbp2*^−/−^ CD133^+^CD44^−^ cells and *Gtpbp2*^+/+^ CD133^+^CD44^−^ cells expressed comparable Ki67, proliferated at the same rate, and showed equivalent viability after 5-FU treatment (Supplementary Figs. 12F–12I). Additionally, *Gtpbp2*^−/−^ CD133^+^CD44^−^ cells and *Gtpbp2*^+/+^ CD133^+^CD44^−^ cells expressed comparable total β-catenin and phosphorylated β-catenin, respectively (Supplementary Figs. 12J–12M). Accordingly, GTPBP2 is dispensable for the function of CD133^+^CD44^−^ cells.

### 3.7 *Gtpbp2* knockout down-regulates β-catenin in CCSC

**Fig. 5:**
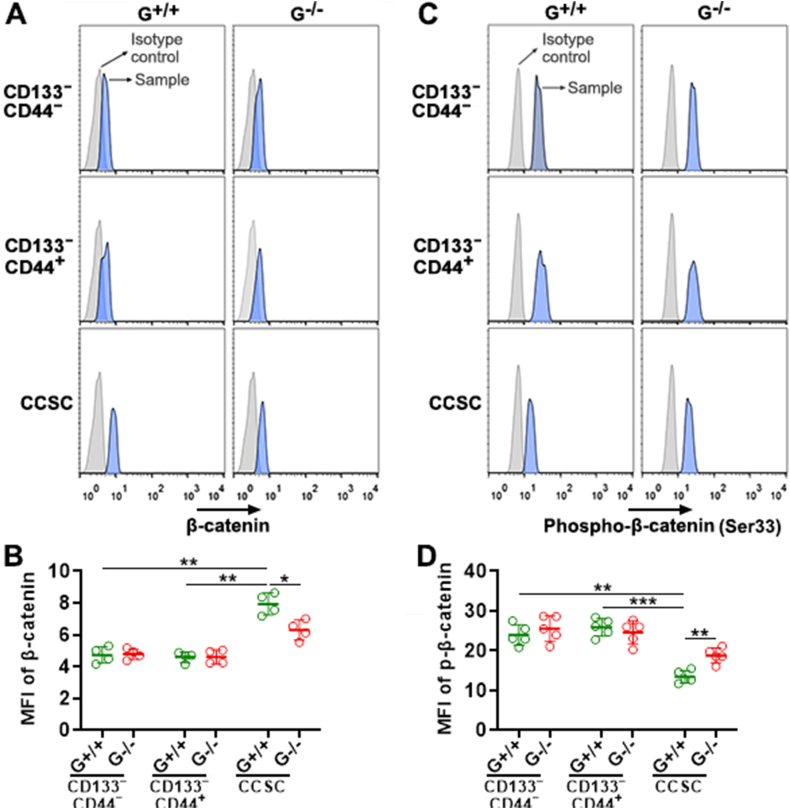
The effect of *Gtpbp2* knockout on β-catenin expression. (A and B) β-catenin expression in *Gtpbp2*^+/+^ and *Gtpbp2*^−/−^ CRC cell subsets on post-infection day 2. Representative histograms are in (A). Statistics of the mean fluorescence of β-catenin are in (B). G^+/+^: *Gtpbp2*^+/+^ cells. G^−/−^: *Gtpbp2*^−/−^ cells. **(C and D)** Phosphorylated β-catenin on post-infection day 2. Representative histograms are in (C). Statistics of the mean fluorescence of phosphorylated β-catenin are in (D). N = 4 or 5 independent samples (no replicates) per group. *: *P* < 0.05. **: *P* < 0.01. ***: *P* < 0.001. One-way ANOVA.

To determine whether GTPBP2 is involved in canonical Wnt signaling, we quantified β-catenin expression in *Gtpbp2*^+/+^ cells and *Gtpbp2*^−/−^ cells, respectively. As shown in Fig. 5A and B, *Gtpbp2*^+/+^ CCSCs expressed higher β-catenin than *Gtpbp2*^+/+^ CD133^−^CD44^−^ and *Gtpbp2*^+/+^ CD133^−^CD44^+^ cells. *Gtpbp2*^+/+^ CCSCs also expressed higher β-catenin than *Gtpbp2*^−/−^ CCSCs. Since β-catenin phosphorylation induces β-catenin degradation, we then quantified phosphorylated β-catenin in each subset. As shown in Fig. 5C and D, *Gtpbp2*^+/+^ CCSCs expressed less phosphorylated β-catenin than *Gtpbp2*^+/+^ CD133^−^CD44^−^ and *Gtpbp2*^+/+^ CD133^−^CD44^+^ cells. Notably, *Gtpbp2*^−/−^ CCSCs expressed higher phosphorylated β-catenin than *Gtpbp2*^+/+^ CCSCs. Hence, GTPBP2 maintained β-catenin in CCSC via suppressing β-catenin phosphorylation.

### 3.8 Wnt agonist SKL2001 abolishes the effects of *Gtpbp2* knockout

**Fig. 6:**
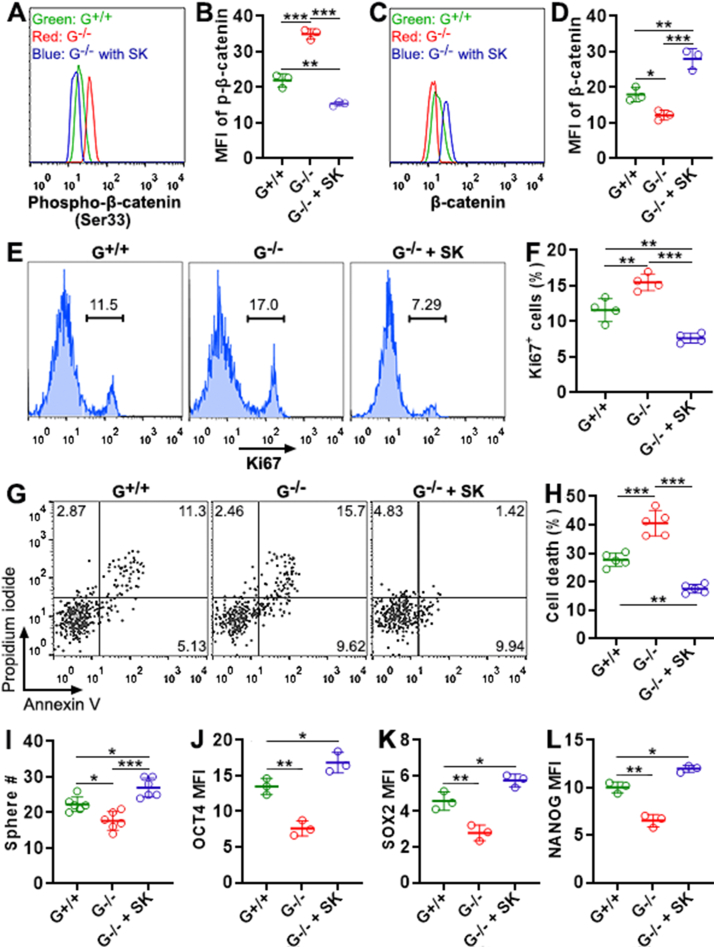
The effect of Wnt agonist SKL2001 on *Gtpbp2*^−/−^ CCSCs. (A and B) Phosphorylated β-catenin after *Gtpbp2*^−/−^ CCSCs were treated with 20 μM SKL2001 for 24 h. G^+/+^: *Gtpbp2*^+/+^ CCSCs. G^−/−^: *Gtpbp2*^−/−^ CCSCs. G^−/−^ with SK: *Gtpbp2*^−/−^ CCSCs treated with SKL2001. Representative histograms are in (A). Statistics of the mean fluorescence of phosphorylated β-catenin are in (B). **(C and D)** β-catenin expression after *Gtpbp2*^−/−^ CCSCs were treated with SKL2001 for 24 h. Representative histograms are in (C). Statistics are in (D). **(E and F)** Ki67 expression after *Gtpbp2*^−/−^ CCSCs were treated with SKL2001 for 24 h. Representative histograms are in (E). Statistics are in (F). **(G and H)** Apoptosis and necrosis of *Gtpbp2*^−/−^ CCSCs in the presence of SKL2001 and 5-FU. Representative dot plots are in (G). Statistics of total cell death (apoptosis + necrosis) are in (H). **(I)** Passage-2 sphere formation. **(J to L)** The mean fluorescence of OCT4, SOX2, and NANOG in *Gtpbp2*^−/−^ CCSCs. N = 3 to 6 independent samples (**no replicate**s) per group in (B, D, F, H, and I). Three samples in duplicate were shown in each group in (J). *: *P* < 0.05. **: *P* < 0.01. ***: *P* < 0.001. One-way ANOVA.

To substantiate the relationship between Wnt signaling and GTPBP2, we treated *Gtpbp2*^−/−^ CCSCs with SKL2001 (a selective Wnt agonist that prevents β-catenin degradation) for 24 h. In SKL2001-treated *Gtpbp2*^−/−^ CCSCs, phosphorylated β-catenin was profoundly decreased to a level lower than that in *Gtpbp2*^+/+^ CCSCs (Fig. 6A and B). Consistently, total β-catenin in SKL2001-treated *Gtpbp2*^−/−^ CCSCs became higher than that in *Gtpbp2*^+/+^ CCSCs (Fig. 6C and D). SKL2001-treated *Gtpbp2*^−/−^ CCSCs had fewer Ki67^+^ cells than *Gtpbp2*^+/+^ CCSCs, suggesting that they were less proliferative (Fig. 6E and F). After 5-FU exposure, SKL2001-treated *Gtpbp2*^−/−^ CCSCs exhibited fewer dead cells than *Gtpbp2*^+/+^ CCSCs, implying that they were more chemoresistant (Fig. 6G to H). At passage 2, SKL2001-treated *Gtpbp2*^−/−^ CCSCs generated more spheres than *Gtpbp2*^+/+^ CCSCs, suggesting stronger self-renewal (Fig. 6I). SKL2001-treated *Gtpbp2*^−/−^ CCSCs expressed higher OCT4, SOX2, and NANOG than *Gtpbp2*^+/+^ CCSCs (Fig. 6J–L and Supplementary Fig. 13). Therefore, SKL2001 enhanced stemness of *Gtpbp2*^−/−^ CCSCs.

### 3.9 *Gtpbp2* knockout in CCSCs results in consistent changes *in vivo*

**Fig. 7:**
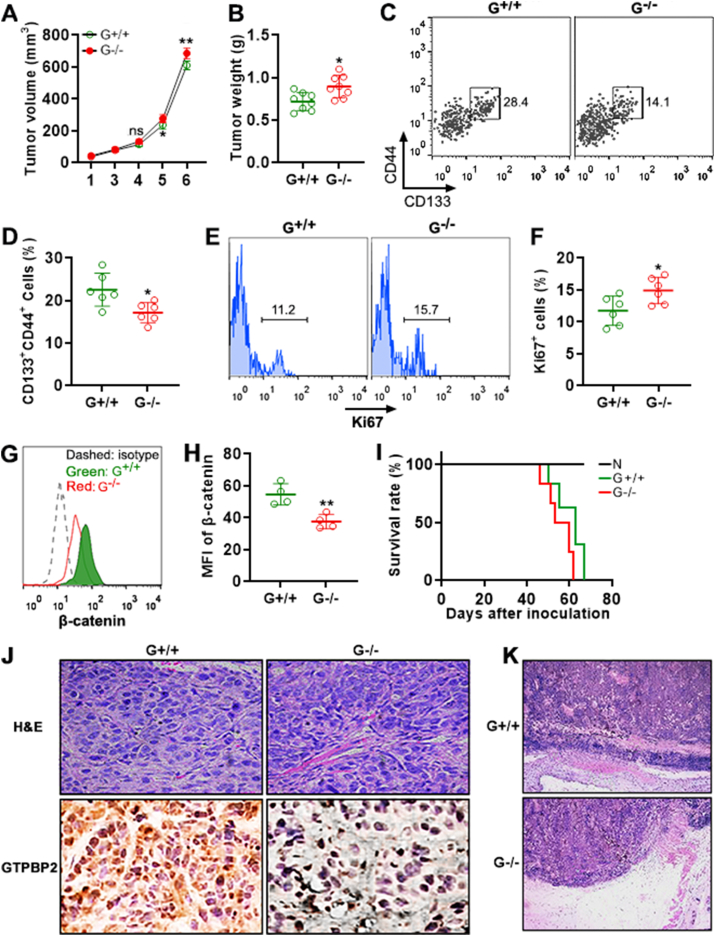
*Gtpbp2* knockout effects *in vivo*. (A) Subcutaneous tumor volume. G^+/+^: *Gtpbp2*^+/+^ CCSC-derived tumor. G^−/−^: *Gtpbp2*^−/−^ CCSC-derived tumor. **(B)** Tumor weight at week 6. **(C and D)** Frequencies of CD133^+^CD44^+^ cells in *Gtpbp2*^+/+^ CCSC-derived tumor cells and *Gtpbp2*^−/−^ CCSC-derived tumor cells, respectively. Representative dot plots are in (C). Statistics are in (D). **(E and F)** Ki67 expression in *Gtpbp2*^+/+^ CCSC-derived or *Gtpbp2*^−/−^ CCSC-derived CD133^+^CD44^+^ cells. Representative histograms are in (E). Statistics are in (F). **(G and H)** β-catenin expression in *Gtpbp2*^+/+^ CCSC-derived or *Gtpbp2*^−/−^ CCSC-derived CD133^+^CD44^+^ cells. Representative histograms are in (G). Statistics are in (H). **(I)** Survival curve of *Gtpbp2*^+/+^ CCSC-inoculated mice or *Gtpbp2*^−/−^ CCSC-inoculated mice. N = 4 to 10 mice (each dot represents an individual mouse) per group. *: *P* < 0.05. **: *P* < 0.01. Student's *t*-test. **(J)** Histological analysis of tumor implants derived from *Gtpbp2*^+/+^ CCSCs and *Gtpbp2*^−/−^ CCSCs. Upper panel: H&E staining. Lower panel: GTPBP2 staining in tumors. Original magnification = 400 × . **(K)** Representative H&E staining images showing the edges of tumor implants. Original magnification = 100 × .

To test the role of GTPBP2 *in vivo*, *Gtpbp2*^+/+^ CCSCs and *Gtpbp2*^−/−^ CCSCs were subcutaneously implanted in nude mice, respectively. Tumor implant growth was monitored every week. *Gtpbp2*^−/−^ CCSCs generated larger tumors than *Gtpbp2*^+/+^ CCSCs from week 5 (Fig. 7A and Supplementary Fig. 14). Consistently, *Gtpbp2*^−/−^ CCSC-derived tumors were heavier than *Gtpbp2*^+/+^ CCSC-derived tumors (Fig. 7B). The tumors were then processed to enrich CD45^−^CD31^−^MCT4^-^ tumor cells as described in Fig. 1. We found fewer CD133^+^CD44^+^ cells (17.2%) in *Gtpbp2*^−/−^ CCSC-derived tumor cells in comparison to *Gtpbp2*^+/+^ CCSC-derived tumor cells (22.6%) (Fig. 7C and D), suggesting weaker self-renewal of *Gtpbp2*^−/−^ CCSCs. Furthermore, *Gtpbp2*^−/−^ CCSC-derived CD133^+^CD44^+^ cells expressed higher Ki67 than *Gtpbp2*^+/+^ CCSC-derived CD133^+^CD44^+^ cells, indicating that they were more proliferative (Fig. 7E and F). *Gtpbp2*^−/−^ CCSC-derived CD133^+^CD44^+^ cells expressed less β-catenin than *Gtpbp2*^+/+^ CCSC-derived CD133^+^CD44^+^ cells (Fig. 7G and H). Mice inoculated with *Gtpbp2*^−/−^ CCSCs survived shorter (average survival period = 53.6 days) than mice inoculated with *Gtpbp2*^+/+^ CCSCs (average survival period = 60.4 days) (Fig. 7I). The histological features of *Gtpbp2*^+/+^ CCSC-derived tumors and *Gtpbp2*^−/−^ CCSC-derived tumors seemed similar (Fig. 7J). GTPBP2 expression was positive in *Gtpbp2*^+/+^ CCSC-derived tumors but negative in *Gtpbp2*^−/−^ CCSC-derived tumors (Fig. 7J). No significant tumor cell invasion into adjacent tissues was seen in either *Gtpbp2*^+/+^ CCSC-derived tumors or *Gtpbp2*^−/−^ CCSC-derived tumors (Fig. 7K).

## 4 Discussion

CCSCs fuel CRC growth, metastasis, relapse, and chemoresistance, rendering them a promising therapeutic target [25]. GTPBP2 belongs to the G protein superfamily. G proteins, capable of binding guanosine triphosphate (GTP) and guanosine diphosphate (GDP), act as molecular switches and signal transducers inside cells [26]. The role of GTPBP2 in either normal cellular processes or malignant transformation is largely unknown. GTPBP2-deficient mice do not exhibit remarkable phenotypic anomalies [15]. In *Xenopus* embryos, GTPBP2 was reported to interact with Axin to positively regulate Wnt signaling and participate in BMP/SMAD1 signaling [18,27]. Considering the essential role of the conserved Wnt signaling in CCSC biology, it is plausible to deduce that GTPBP2 might regulate Wnt signaling in CCSCs.

We enriched primary CCSC according to the expression of CD133 and CD44, which are considered valid CCSC markers in human patients and mice [1,22,28]. However, the distribution of CCSC markers differs between patients and tumor cell lines. For example, CD133^−^CD44^+^ cells correlate with CCSC features in the SW620 cell line and CRC patients [6,8]. CD133^high^CD24^low^ cells are probably CCSC in patients with CRC since they are associated with the worst prognosis [7]. Therefore, the universal set of markers to identify CCSCs in CRC remains ambiguous. Our data indicated that CD133^−^CD44^+^ cells did not express abundant stemness markers and failed to generate spheres, implying that these cells were unlikely CCSCs in our experimental setting. In our study, CD133^+^CD44^+^ CCSCs expressed higher GTPBP2, signifying the potential importance of this molecule in CCSC activity. However, the cause of GTPBP2 up-regulation is not clear. To our knowledge, no previous study has delineated the transcriptional or translational mechanisms of GTPBP2 expression. Therefore, future investigations are necessary to reveal whether stemness-associated signaling pathways, such as the Hedgehog pathway and Notch pathway, are responsible for GTPBP2 up-regulation. Moreover, it would be interesting to check whether normal colonic stem cells, i.e. stem cells in the bottom of the colonic crypt [29], have high GTPBP2 expression.

Intriguingly, GTPBP2 ablation significantly altered the proliferation, chemoresistance, and self-renewal of CCSCs rather than CD133^−^CD44^−^ (Subset I) and CD133^−^CD44^+^ (Subset II) CRC cells, implying that GTPBP2 is essential for CCSC activity but not differentiated CRC cells. GTPBP2 is probably not important for differentiated CRC cell growth due to its low expression in Subset I and Subset II. Another possibility is that GTPBP2 deficiency might be compensated by other G proteins or pathways in differentiated CRC cells. Therefore, the remarkable role of GTPBP2 seems to be CCSC-specific.

Our data indicate that GTPBP2 ablation accelerates CCSC division but impairs CCSC self-renewal. There are two types of stem cell division: symmetric division and asymmetric division. The former means that a stem cell produces two identical stem cells, while the latter indicates that a stem cell produces one differentiated cell and one stem cell [30]. Therefore, symmetric division is more efficient in maintaining the self-renewal capacity, whereas asymmetric division incurs a gradual decrease in the self-renewal capacity of a group of stem cells. Although we did not assess the types of *Gtpbp2*^−/−^ and *Gtpbp2*^+/+^ CCSCs division, the weaker sphere formation of *Gtpbp2*^−/−^ CCSCs suggests that GTPBP2 ablation promotes asymmetric CCSC division and subsequently diminishes CCSCs’ self-renewal capacity. Moreover, the slower division of *Gtpbp2*^+/+^ CCSCs indicates that GTPBP2 is essential for CCSC quiescence that reinforces chemoresistance. Therefore, the higher resistance of *Gtpbp2*^+/+^ CCSCs to 5-FU could be attributed to the quiescent status. In the future, bulk or single-cell transcriptome sequencing should be performed to answer whether *Gtpbp2*^−/−^ and *Gtpbp2*^+/+^ CCSCs differentially express self-renewal- or quiescence-associated genes. A recent study applied single-cell whole-exome sequencing and bulk-cell targeted exome sequencing to demonstrate that CD133^+^CD44^−^ cells are likely the original CCSC population [8]. However, in another recent research, transcriptome profiling of human CRC samples by SMART-seq2 indicates that CSCs are enriched from both CD44^+^CD133^+^ and CD44^−^CD133^+^ CRC cells [31]. These studies exhibit the complexity and heterogeneity of CCSCs and provide valuable data to analyze the stemness and quiescence of putative CCSCs.

Another important finding is the down-regulation of Wnt signaling in Gtpbp2^−/−^ CCSCs, which is consistent with a previous study reporting that GTPBP2 positively regulates Wnt signaling [18]. Wnt signaling activation enhances stemness, self-renewal, and chemoresistance [11,12,32,33]. Therefore, GTPBP2 ablation attenuates Wnt signaling activation and consequently harms the self-renewal and chemoresistance of CCSCs. Regarding cell proliferation, although Wnt signaling appears to promote the proliferation of CRC cells [10], it enforces the quiescence of hematopoietic stem cells and neural stem cells [34,35]. Furthermore, SKL2001 facilitates colon cancer spheroid formation but suppresses spheroid growth by inducing G0/G1 phase arrest, implying that the Wnt signaling facilitates CCSC self-renewal but inhibits CCSC proliferation [36]. In our study, SKL2001 also inhibited CCSC proliferation, strongly indicating that Wnt signaling maintains CCSC quiescence. SKL2001 disrupts the interaction between β-catenin and Axin to prevent β-catenin phosphorylation and subsequent degradation. Indeed, we observed lower β-catenin phosphorylation and elevated β-catenin levels in SKL2001-treated *Gtpbp2*^−/−^ CCSCs. Although we did not perform co-immunoprecipitation to determine the potential interaction between GTPBP2 and the destruction complex owing to the rarity of primary CCSCs, this assay could be done on human or mouse CRC cell lines in the future.

Metastatic CRC cells release proteolytic enzymes like matrix metalloproteinase 2 (MMP-2) and matrix metalloproteinase 9 (MMP-9) to digest the extracellular matrix (ECM), facilitating irregular cancer cords to penetrate adjacent tissues [37]. Other factors, such as E-cadherin down-regulation, N-cadherin up-regulation, and enhanced motility of cancer cells also contribute to CRC invasion [38]. Previous research has suggested that the Wnt signaling might promote the expression of MMP-2 and MMP-9 in liver cancer [39]. Besides, the Wnt signaling increases the invasion of CRC [40]. Therefore, GTPBP2-induced β-catenin stabilization would reinforce CRC invasion. However, our *in vitro* and *in vivo* data imply that GTPBP2 does not affect CRC cell migration or invasiveness. Perhaps GTPBP2 activates other signal pathways to suppress β-catenin-mediated invasiveness. This puzzle needs to be unraveled in future investigations.

Apart from the above findings, we found that stemness markers such as OCT4, SOX2, and NANOG were highly expressed in CCSCs relative to other subpopulations. GTPBP2 ablation only down-regulated these markers in CCSCs, strongly implying that GTPBP2 up-regulates the expression of stemness markers in CCSCs. Among these markers, NANOG is a transcription factor key to the stemness of cancer stem cells because it induces self-renewal, invasiveness, and chemoresistance of cancer cells through multiple pathways [41]. Importantly, NANOG induces the dormancy, migration, and chemoresistance of colorectal cancer cells [42,43]. Therefore, GTPBP2 likely activates the Wnt signaling to induce high NANOG expression, and NANOG promotes CCSC stemness and maintains the malignant nature of CCSCs. However, GTPBP2 ablation or overexpression could not affect the expression of NANOG and other stemness markers in CD133^−^CD44^−^ and CD133^−^CD44^+^ cells, suggesting that GTPBP2 no longer regulates stemness in non-CCSC cancer cells probably due to altered intracellular microenvironment.

In summary, our study demonstrates that GTPBP2 positively modulates Wnt signaling to reinforce the quiescence, self-renewal, and chemoresistance of CCSCs. We disclose a novel mechanism underlying CCSC biology and GTPBP2 could be a therapeutic target in future CRC treatment.

## Ethics approval

This animal research was approved by the Wuhan Third Hospital Animal Care and Use Committee (Approval ID: WTH20210146) and was conducted under the Animal Research: Reporting of In Vivo Experiments (ARRIVE) guidelines.

## Funding

This study was supported by the Foundation for Scientific Research Projects from Wuhan Municipal Health Commission (Grant# WX20A07).

## Data availability statement

Data are available from the corresponding author upon reasonable requests and with permission from the institute.

## CRediT authorship contribution statement

**Chao Ke:** Methodology, Investigation, Funding acquisition. **Hongjian Zhou:** Investigation. **Tian Xia:** Methodology, Investigation. **Xingwang Xie:** Validation, Conceptualization. **Bin Jiang:** Writing – review & editing, Writing – original draft, Supervision, Project administration, Methodology, Data curation, Conceptualization.

## Declaration of competing interest

The authors declare that they have no known competing financial interests or personal relationships that could have appeared to influence the work reported in this paper.
