# Supplementary material for: GTP binding protein 2 maintains the quiescence, self-renewal, and chemoresistance of mouse colorectal cancer stem cells via promoting Wnt signaling activation
Source: Heliyon. 2024 Mar 1;10(5):e27159. doi: 10.1016/j.heliyon.2024.e27159 (PMC10926081; doi:10.1016/j.heliyon.2024.e27159)
Supplement: Multimedia component 1 [file mmc1.docx]

| **Supplementary Table 1. Primers** | | |
| --- | --- | --- |
| **Target** | **Forward (5’-3’)** | **Reverse (5’-3’)** |
| **Cyclin D1 (*Ccnd1*)** | GCAGAAGGAGATTGTGCCATCC | AGGAAGCGGTCCAGGTAGTTCA |
| **Cyclin E1 (*Ccne1*)** | AAGCCCTCTGACCATTGTGTCC | CTAAGCAGCCAACATCCAGGAC |
| **Cyclin A1 (*Ccna1*)** | GCTACTGAGGATGGAGCATCTG | CAGCTTCCAGAAGGCTCAGTTC |
| **Cyclin B1 (*Ccnb1*)** | AGAGGTGGAACTTGCTGAGCCT | GCACATCCAGATGTTTCCATCGG |
| **OCT4 (*Pou5f1*)** | CAGCAGATCACTCACATCGCCA | GCCTCATACTCTTCTCGTTGGG |
| **SOX2 (*Sox2*)** | AACGGCAGCTACAGCATGATGC | CGAGCTGGTCATGGAGTTGTAC |
| **NANOG (*Nanog*)** | GAACGCCTCATCAATGCCTGCA | GAATCAGGGCTGCCTTGAAGAG |
| **GTPBP2 (*Gtpbp2*)** | GGACTGTGGTTGGAGGAACACT | ACCTGCTCGAAGAACACGACAC |
| **β-actin (*Actb*)** | CATTGCTGACAGGATGCAGAAGG | TGCTGGAAGGTGGACAGTGAGG |


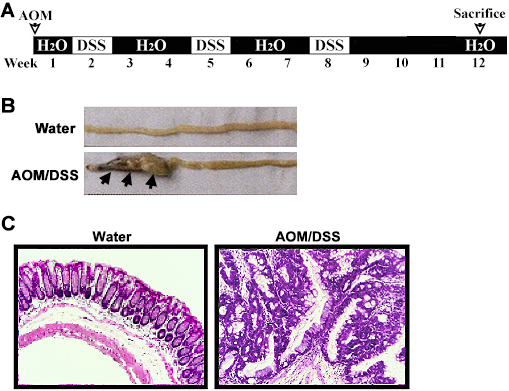


**Supplementary Figure 1. CRC induction. (A)** Schematic diagram of AOM plus DSS treatment. **(B)** Gross specimen photographs of tumor formation in the colon and rectum at month 3 (week 12). Black arrows indicate tumors. **(C)** H&E staining of normal mouse colon (Water) and CRC formation after AOM/DSS treatment (Original magnification: 100×).


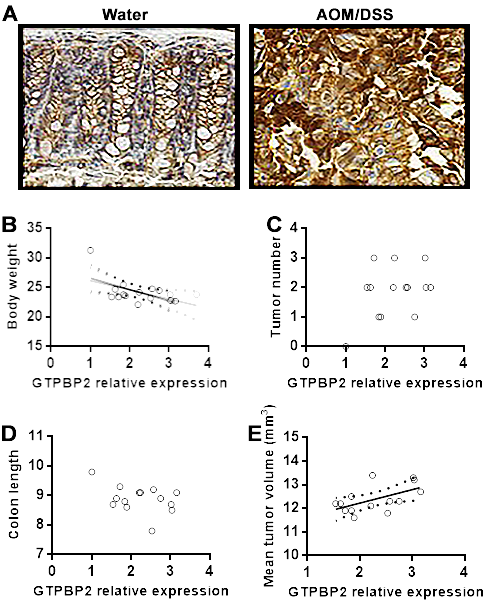


**Supplementary Figure 2. GTPBP2 expression in CRC. (A)** Immunohistochemistry of GTPBP2 expression in normal mouse colon (Water) and a CRC tumor after AOM/DSS treatment (Original magnification: 200×). **(B to E)** The correlations between GTPBP2 mRNA level and mouse body weight (B), tumor number (C), colon length (D), and mean tumor volume (E). “1” in the X-axis indicates GTPBP2 expression in the normal colon. GTPBP2 relative expression level (0-4) was calculated by real-time RT-PCR in the following steps:

1. Normalized GTPBP2 level:

ΔCt1= Ct_(GTPBP2 in CRC mass)_ - Ct_(β-actin in CRC mass)_

ΔCt2= Ct_(GTPBP2 in normal colon)_ - Ct_(β-actin in normal colon)_

1. GTPBP2 relative expression level = 2^-(ΔCt1- ΔCt2)^


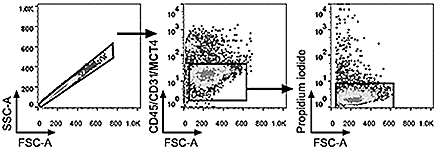


**Supplementary Figure 3. Sequential gating of CD45^-^CD31^-^MCT4^-^ live cells among single cells isolated from CRC tumors.**


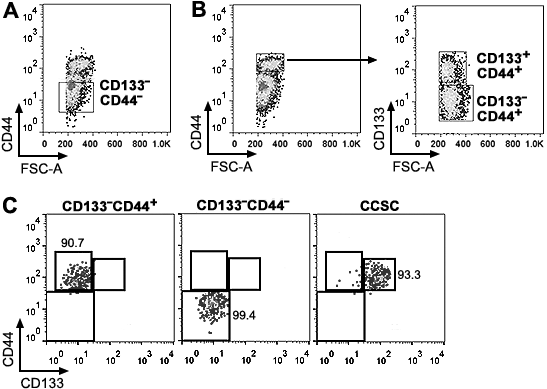


**Supplementary Figure 4. Sorting CRC cell subsets. (A)** Sorting CD133^-^CD44^-^ cells. **(B)** Gating CD44^+^ cells and then sorting CD133^+^CD44^+^ (CCSCs) and CD133^-^CD44^+^ cells. **(C)** The purity of sorted CD133^-^CD44^+^, CD133^-^CD44^-^, and CD133^+^CD44^+^ cells (CCSCs), respectively.


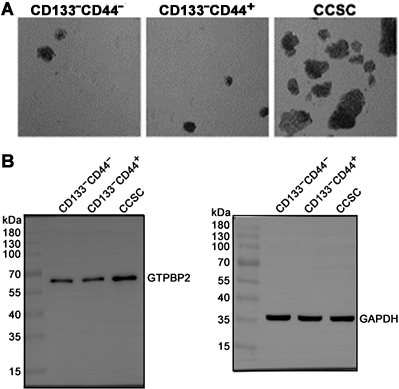


**Supplementary Figure 5. GTPBP2 protein expression in sorted CRC cell subsets. (A)** Passage-1 tumor spheres formed by sorted CD133^-^CD44^-^, CD133^-^CD44^+^, and CD133^+^CD44^+^ cells (CCSCs). Original magnification: 50×. **(B)** Uncropped images of Figure 1B.


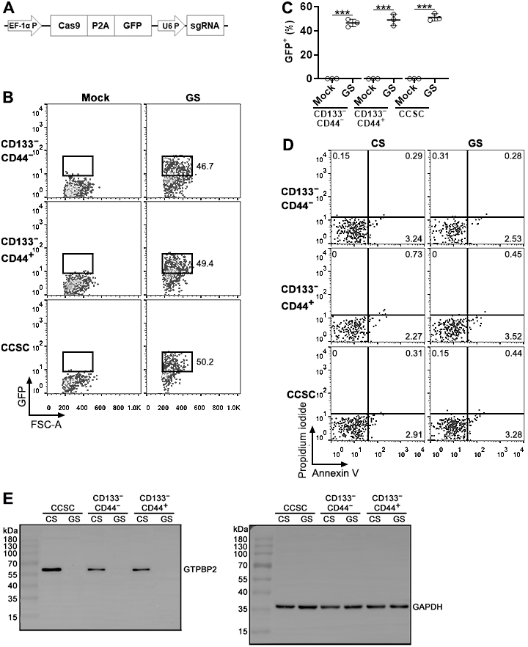


**Supplementary Figure 6. Lentivirus infection of sorted CRC cells. (A)** The map of the all-in-one Cas9/sgRNA lentivector. **(B)** Dot plots showing GFP expression in CRC cells on post-infection day 2. Mock: transduction reagents without lentivirus. GS: lentivirus encoding the *Gtpbp2* sgRNA. **(C)** Frequencies of GFP^+^ cells on post-infection day 2. N= 3 independent samples in 3 individual experiments. ***: *P*<0.001. **(D)** Dot plots showing apoptosis and necrosis of GFP^+^ cells on post-infection day 2. CS: control lentivirus encoding a scrambled sgRNA. GS: lentivirus encoding the *Gtpbp2* sgRNA. This image represents two independent experiments. **(E)** Uncropped images of Figure 2A.


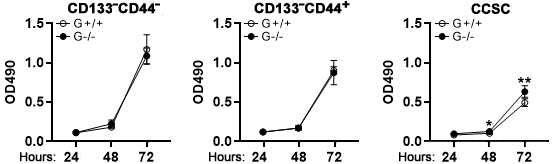


**Supplementary Figure 7. Growth curves (OD490 values) of CD133^-^CD44^-^ cells, CD133^-^CD44^+^ cells, and CCSCs.** G^+/+^: *Gtpbp2*^+/+^ cells. G^-/-^: *Gtpbp2*^-/-^ cells. N=6 samples (3 independent samples in duplicate) per time point per group. *: *P*<0.05 (G^+/+^ vs G^-/-^). ***: *P*<0.001 (G^+/+^ vs G^-/-^).


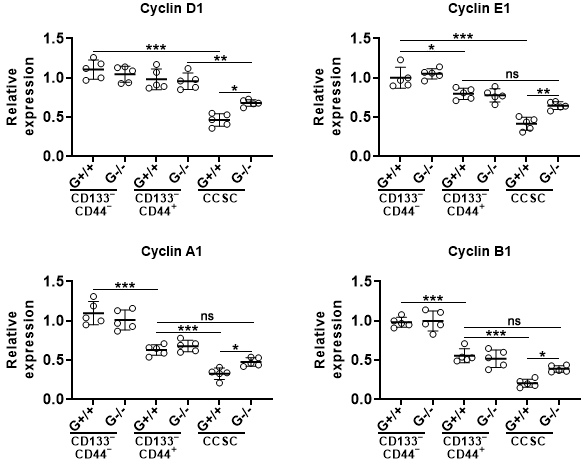


**Supplementary Figure 8. mRNA levels of indicated Cyclins in CD133^-^CD44^-^ cells, CD133^-^CD44^+^ cells, and CCSCs.** G^+/+^: *Gtpbp2*^+/+^ cells. G^-/-^: *Gtpbp2*^-/-^ cells. N=5 independent samples (**no replicates**) per group. *: *P*<0.05. **: *P*<0.01. ***: *P*<0.001. ns: not significant.


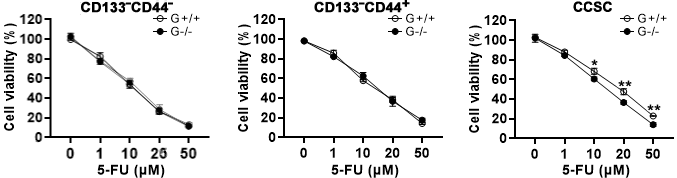


**Supplementary Figure 9. Cell viability after treatment with indicated concentrations of 5-FU for 48 hours.** G^+/+^: *Gtpbp2*^+/+^ cells. G^-/-^: *Gtpbp2*^-/-^ cells. N=6 samples (2 independent samples in triplicate) per group. *: *P*<0.05 (G^+/+^ vs G^-/-^). **: *P*<0.01 (G^+/+^ vs G^-/-^).


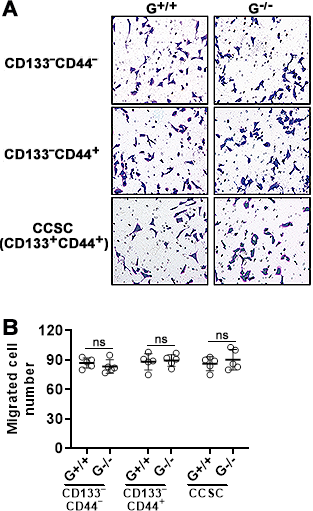


**Supplementary Figure 10. Cell migration. (A)** Representative images showing migrated CD133^-^CD44^-^ cells, CD133^-^CD44^+^ cells, and CCSCs on the lower side of the membranes. G^+/+^: *Gtpbp2*^+/+^ cells. G^-/-^: *Gtpbp2*^-/-^ cells. **(B)** Statistics of migrated cell number. N=5 independent samples (no replicates) per group. ns: non-significant. Student’s t-test.

**
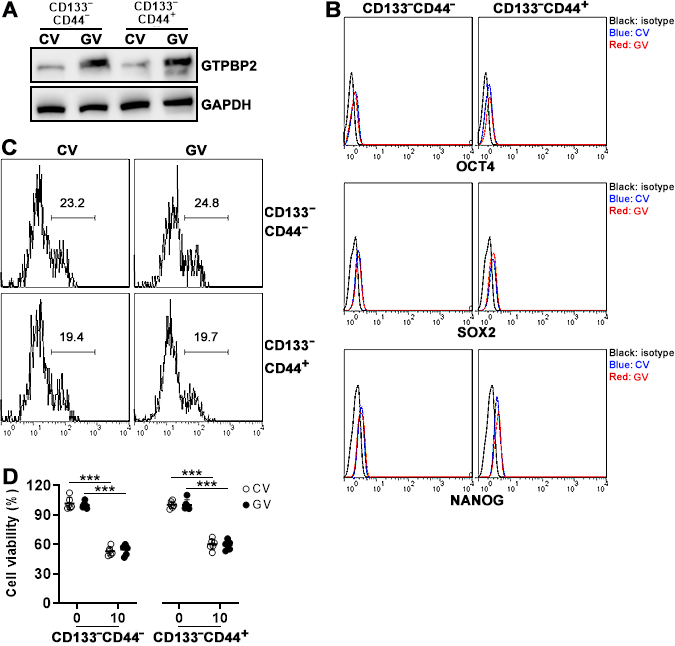
**

**Supplementary Figure 11. The effect of *Gtpbp2* overexpression on CD133^-^CD44^-^ and CD133^-^CD44^+^ cells. (A)** GTPBP2 protein after lentiviral infection. CV: infection with the control empty lentivirus. GV: infection with the *Gtpbp2*-encoding lentivirus. The images represent two independent experiments. **(B)** Representative histograms showing the expression of OCT4, SOX2, and NANOG. The images represent two independent experiments. **(C)** Representative histograms showing Ki67 expression in infected cells. The images represent two independent experiments. **(D)** Cell viability after 10 μM 5-FU treatment. N=6 samples (2 independent samples in triplicate) per group. ***: *P*<0.001.


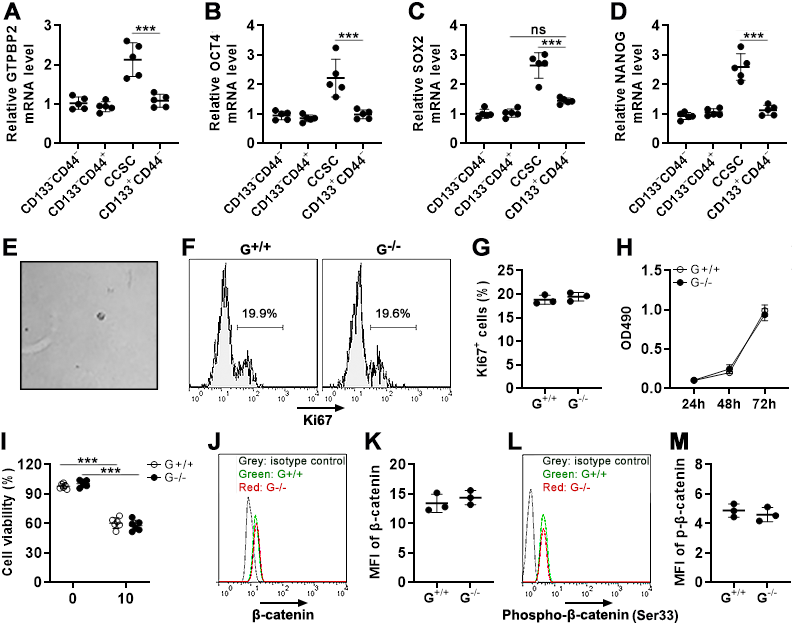


**Supplementary Figure 12. The effect of GTPBP2 on the phenotype and function of CD133^+^CD44^-^ cells. (A to D)** mRNA levels of GTPBP2, OCT4, SOX2, and NANOG in CD133^-^CD44^-^ cells, CD133^-^CD44^+^ cells, CCSCs, and CD133^+^CD44^-^ cells. **(E)** A representative image showing no sphere formation from sorted CD133^+^CD44^-^ cells. **(F)** Representative histograms showing Ki67 expression after *Gtpbp2* knockout. G^+/+^: *Gtpbp2*^+/+^ CD133^+^CD44^-^ cells. G^-/-^: *Gtpbp2*^-/-^ CD133^+^CD44^-^ cells. **(G)** Statistics of the frequencies of Ki67^+^ cells. **(H)** Growth curves of *Gtpbp2*^+/+^ CD133^+^CD44^-^ cells and *Gtpbp2*^-/-^ CD133^+^CD44^-^ cells. **(I)** Cell viability after 5-FU treatment. **(J)** Representative histograms showing total β-catenin expression. **(K)** Statistics of the mean fluorescence of total β-catenin. **(L)** Representative histograms showing phosphorylated β-catenin expression. **(M)** Statistics of the mean fluorescence of phosphorylated β-catenin. N=3 to 6 independent samples per group. ***: *P*<0.001. ns: not significant.


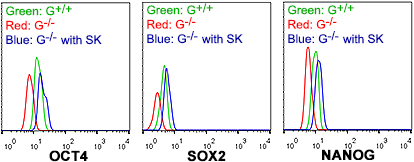


**Supplementary Figure 13. Representative histograms showing the expression of OCT4, SOX2, and NANOG in *Gtpbp2*^-/-^ CCSCs after treatment with 20 μM SKL2001 for 24 hours.** G^+/+^: *Gtpbp2*^+/+^ CCSCs. G^-/-^: *Gtpbp2*^-/-^ CCSCs. G^-/-^ with SK: *Gtpbp2*^-/-^ CCSCs treated with SKL2001. The statistics are shown in Figure 6J to 6L.


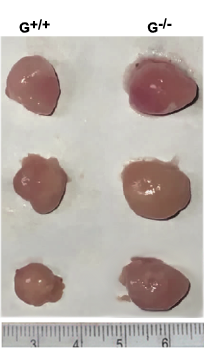


**Supplementary Figure 14. Tumor implants harvested at week 6 from recipient nude mice.** G^+/+^: G*tpbp2*^+/+^ CCSC-derived tumor. G^-/-^: G*tpbp2*^-/-^ CCSC-derived tumor.
